# A Jasmonate-Induced Defense Elicitation in Mature Leaves Reduces Carbon Export and Alters Sink Priority in Grape (*Vitis vinifera* Chardonnay)

**DOI:** 10.3390/plants10112406

**Published:** 2021-11-08

**Authors:** Nick Gould, Michael R. Thorpe, Joe T. Taylor, Helen L. Boldingh, Catherine M. McKenzie, Tony Reglinski

**Affiliations:** 1 The New Zealand Institute for Plant and Food Research Limited, 412 No 1 Road, RD 2, Te Puke 3182, New Zealand; catherine.mckenzie@plantandfood.co.nz; 2 IBG-2: Plant Sciences, Forschungszentrum Jülich, D-52425 Jülich, Germany; michael.thorpe@anu.edu.au; 3 The New Zealand Institute for Plant and Food Research Limited, Ruakura, Bisley Road, Hamilton 3214, New Zealand; Joe.taylor@plantandfood.co.nz (J.T.T.); helen.boldingh@plantandfood.co.nz (H.L.B.); tony.reglinski@plantandfood.co.nz (T.R.)

**Keywords:** carbon partitioning, jasmonate, jasmonic acid, methyl jasmonate, plant defence, sink, sink priority, source

## Abstract

This work aims to understand how *Vitis vinifera* (Chardonnay) vines prioritise the export and distribution of recently fixed photoassimilate between root tissue, fruit, and defence, following the elicitation of a defence response. Jasmonic acid (JA) and its methyl ester, MeJA, are endogenous plant hormones, known collectively as jasmonates, that have signalling roles in plant defence and consequently are often used to prime plant defence systems. Here, we use exogenous jasmonate application to mature source leaves of Chardonnay grapevines to elucidate the prioritisation strategy of carbon allocation between plant defence and growth. Our results demonstrate that jasmonate application to Chardonnay leaves can elicit a defence response to *Botrytis cinerea,* but the effect was localised to the jasmonate-treated area. We found no evidence of a systemic defence response in non-treated mature leaves or young growing tissue. JA application reduced the photosynthetic rate of the treated leaf and reduced the export rate of recently fixed carbon-11 from the leaf. Following JA application, a greater proportion of available recently fixed carbon was allocated to the roots, suggesting an increase in sink strength of the roots. Relative sink strength of the berries did not change; however, an increase in berry sugar was observed seven days after JA treatment. We conclude that the data provide evidence for a “high sugar resistance” model in the mature treated leaves of the vine, since the export of carbon was reduced to ensure an elevated defence response in the treated leaf. The increase in berry sugar concentration seven days after treatment can be explained by the initial prioritisation of a greater portion of the exported carbon to storage in the roots, making it available for remobilisation to the berries once the challenge to defence had passed.

## 1. Introduction

Jasmonic acid (JA) and its methyl ester, MeJA, are endogenous plant hormones, known collectively as jasmonates, that are believed to have signalling roles in plant defence against both insects and some necrotrophic pathogens, with JA accumulating in challenged tissues, which can then be rapidly metabolised into MeJA [1,2,3,4]. Furthermore, jasmonates are potential activators that induce a number of anatomical and chemical changes related to plant defence when applied exogenously [5,6]. However, jasmonates can also affect carbon assimilation and partitioning. Jasmonates have been shown to reduce leaf carbon pools by affecting the control of shoot and root growth, reduce expression of genes involved in photosynthesis [7,8,9], and decrease chlorophyll content [7,10], and they are believed to have a role in the partitioning of carbon and nitrogen to vegetative sinks [9]. Thus, there is a delicate balance in the dual roles of jasmonates in both plant defence and plant development, and dynamic interplay between these processes can account for the transient changes in growth rate in the weeks following elicitation of a defence response through jasmonate application [11].

The observations of Creelman and Mullet [9] highlight the complexities of inducing plant defence mechanisms and their effects upon productivity. The literature has a number of examples highlighting the contrasting effects that activators, pathogens, and herbivores have upon plant growth and carbon partitioning. For example, some studies show increased partitioning of carbon to storage tissues [12,13,14]. Exogenous application of JA onto mature photosynthesising leaves of Populus increased carbon export from the leaves to the stem and roots within hours of treatment [12], as did feeding on mature Populus leaves by the gypsy moth (*Lymantria dispar* L.) [13]. Additionally, Henkes et al. [14] noted an increase in carbon partitioning to an untreated portion of barley roots in preference to roots treated with JA. These results have been explained as a method of conserving valuable carbohydrate by transporting it away from potential risk, so the carbon can be later remobilised for growth once the threat has passed [11,12].

Other studies show resources preferentially directed to new growth rather than storage. Arnold and Schultz [15] noted JA application to young, developing leaves increased the carbon preferentially partitioned to the sink leaves of Populus. Additionally, feeding by white-marked tussock moth larvae (*Orgyia leucostigma*) on red oak was observed to increase carbon flows to new shoots in preference to root growth or storage [16]. The increase in carbon import into the growing leaves may be required for the young tissue to metabolise defence compounds in response to a systemic signal. For example, the increased carbon flows to the sink leaves observed by Arnold and Schultz were associated with an increase in tannin production in the sink leaves, whereas reducing the carbohydrate supply to emerging kiwifruit leaves reduced phenolic and tannin concentrations and increased *Botrytis* *cinerea* lesion size in the growing leaves [17], carbohydrate availability being vital to the establishment of a defence response [18].

Responding to jasmonate, herbivory, or pathogen challenges can involve partitioning carbon either into new growth or into storage, as highlighted above. However, successful localised plant defence depends upon available resource at the infection site [19]. This strategy may favour a reduction in carbon export from treated source leaves to provide carbohydrate for defence compounds within the challenged source leaf. *Arabidopsis* leaves were more susceptible to *Colletotrium higginsianum* infection when the sucrose availability in infected leaves was limited by either a reduction in the light period available for carbon fixation or in mutants with reduced starch turnover [18]. Such findings support the idea of “high sugar resistance” [19] and observations that some pathogenesis-related genes are sugar inducible [20,21]. *Phytophtora nicotianae* infection in source leaves of *Nicotiana tabacum* increased glucose-6-phosphate dehydrogenase activity, apoplastic invertase activity, and thus, hexose content [22].

There is great potential in stimulating the jasmonic acid pathway to reduce disease in horticultural crops such as grapes. A suitable activator will reduce disease severity, without delaying fruit development or reducing fruit quality at harvest, although the response will depend on how the plant responds in relation to the three models of carbon partitioning described above (i.e., carbon mobilised to storage, new growth, or used for defence in the treated leaf). In grape, exogenous jasmonate treatments can elicit defence responses. MeJA applied to grape berries post-harvest reduced the incidence of *B. cinerea*, the treated berries increasing activity of key defence enzymes chitinase (CHI), *β*-1,3-glucanase (GNS), phenylalanine ammonia-lyase (PAL), polyphenol oxidase (PPO), and peroxidase (POX). Additionally, 1 mM MeJA foliar application increased total soluble solids content, both total phenolics and anthocyanin concentrations, and accelerated ripening in grape berries [23,24]. Beneficial fruit quality characteristics have also been observed with MeJA application in the vineyard. Two foliar applications of MeJA (at véraison and one week later) increased key berry phenolic compounds associated with wine quality [25]. However, a successful defence response often has an associated cost, as resources are directed away from growth and crop production. As highlighted above, jasmonates can reduce leaf carbon pools by affecting control of shoot and root growth and negatively affect the photosynthetic system. Foliar applications of 5 mM MeJA reduced yield in Magenta and Crimson table grapes [24]. The work presented here investigates the physiological responses of jasmonate application onto mature leaves of Chardonnay grapevines, concerning the impact on photoassimilation, the export of carbon and its partitioning between roots and fruit, and the ability of the vine to elicit a defence response.

## 2. Results

**Figure 1:**
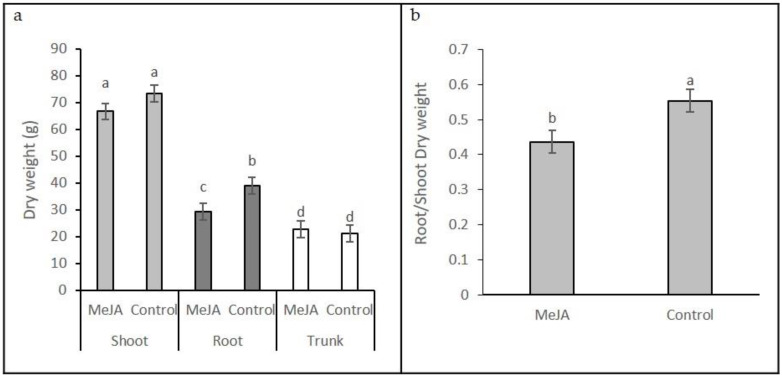
(**a**) Dry weight of shoot, root, and trunk tissue and (**b**) root/shoot dry weight ratio of destructively harvested whole Chardonnay vines following 4-weekly foliar application of 4.5 mM methyl jasmonate (MeJA). Vines were sampled two weeks after the last treatment. Error bars show standard errors. Different letters denote treatments that are significantly different (*n* = 16 vines per treatment; *p* < 0.05).

Trunk, shoot, and root biomass in potted Chardonnay grapevine plants was determined two weeks after the last of 4-weekly foliar MeJA applications. The root dry weight of MeJA-treated vines was significantly reduced (*p* < 0.05; ANOVA) than that in the unreated control, whereas trunk and shoot weights were not different (Figure 1a). The root/shoot dry weight ratio was also significantly reduced in the MeJA-treated vines compared with that of the control vines (*p* < 0.05; ANOVA; Figure 1b).

**Figure 2:**
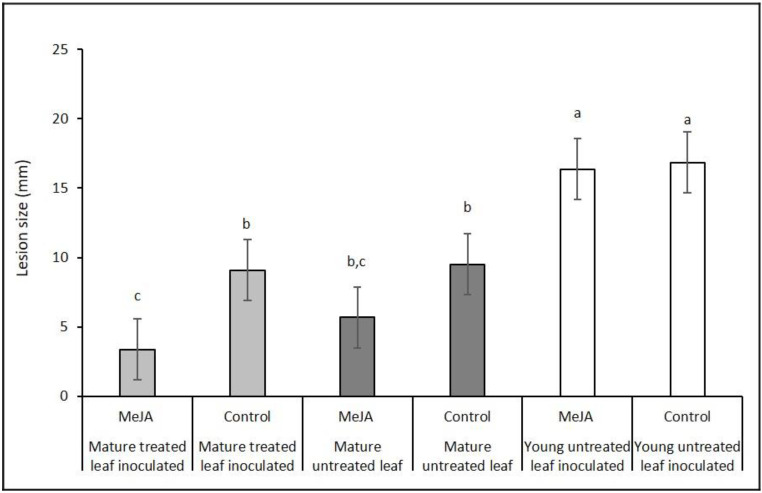
Lesion size five days after mature leaves or young Chardonnay grape leaves were inoculated with *Botrytis cinerea*. For all treatments, a single mature leaf was treated with methyl jasmonate (MeJA) or water (control). The leaves inoculated for each plant were either a mature treated leaf (MeJA or control treated), a mature untreated leaf, or a young, not yet fully expanded untreated leaf. Error bars show standard errors. Bars with the same letters above are not significantly different (*n* = 5 plants, with 3 leaves per plant and 4 inoculations per leaf; *p* < 0.077).

Mature leaves on potted Chardonnay vines developed smaller lesions after inoculation with *B. cinerea* than young leaves (Figure 2). Application of MeJA to a single mature leaf, one week before inoculation, further enhanced resistance to botrytis bunch rot disease (hereafter botrytis) in the treated leaf compared with the equivalent leaf in the control vines treated only with water + Tween^®^ (Figure 2, *p* < 0.077; ANOVA). There was no significant effect of MeJA on botrytis development on untreated leaves, compared with control plants, indicating that the MeJA-induced resistance response was not systemic. Young leaves on both the control and MeJA-treated plants had significantly larger lesions than mature leaves in any of the treatments (*p* < 0.05; ANOVA).

Total soluble sugar content of the berries increased significantly one week after the final JA application on the mature leaf (*p* < 0.05; ANOVA; Table 1). JA treatment did not affect berry concentrations of malic, tartaric, or citric acid. Berry total phenolics, peroxidase (POX), and phenylalanine ammonia lyase (PAL) all increased following JA treatment of mature leaves but were not significantly different from the control values (*p*>0.05; ANOVA; Table 1). Cinnamyl alcohol dehydrogenase (CAD) activity was low in the berry and reduced in the JA-treated vines compared with the control, but again, the difference was not statistically significant.

**Figure 3:**
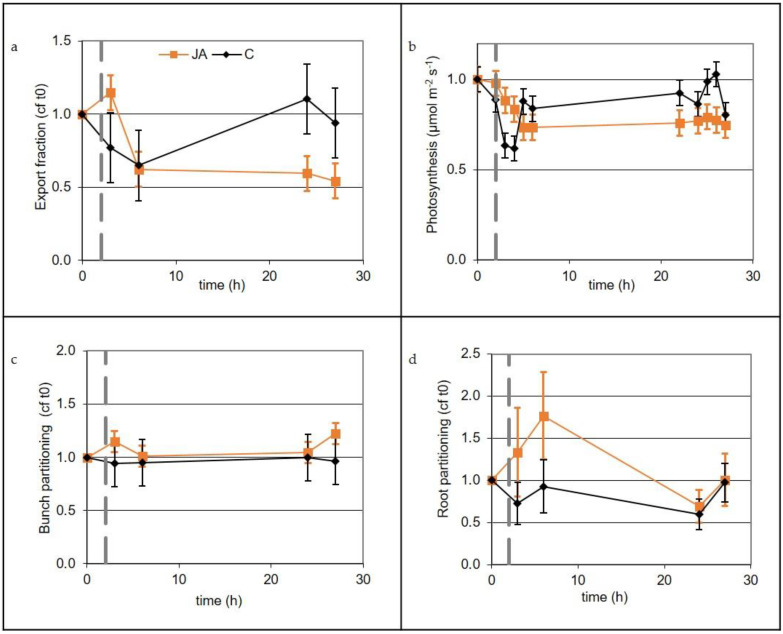
The relative rate of export of recently fixed ^11^C to the initial measurement at *t* = 0 from a mature Chardonnay grape leaf (**a**), photosynthetic rate of the mature leaf (**b**), relative import of available ^11^C into the bunch (**c**), relative import of available ^11^C into the roots (**d**). The mature leaf was treated with 1mM JA at *t* = 2 h indicated by the dotted black line. Means and standard error bars shown, *n* = 6 (jasmonic acid (JA) treated) and *n* = 4 (control).

The export fraction (fraction of recently fixed carbon exported) for a mature source leaf following a single treatment with 1mM JA/Tween^®^, or with water/Tween^®^ for controls, was sampled on five occasions during a 25-hour period from JA application. All statistical analysis for the carbon partitioning and photosynthetic rates was carried out using confidence intervals constructed from mixed model analysis, comparing means with pre-treated values where *t* = 0. Further details are presented in the Section 4. Export from control treated leaves declined over the first 4 h (*p* < 0.05) and then recovered to pre-treatment levels, whereas export in the treated leaves was significantly reduced and remained at a lower value for the duration of the experiment (*p* < 0.05; Figure 3a). The photosynthetic rate in the control and treated leaves reduced in both the JA-treated and control leaves in the three hours following treatment (*p* < 0.05). The rate for control leaves soon recovered to the initial rate, but for JA-treated leaves, the rate remained reduced for at least 25 h after treatment (*p* < 0.05; Figure 3b). Bunch partitioning (the fraction of the exported carbon delivered to the bunch) showed no effect of JA (i.e., bunch partitioning was similar in control and treated plants; Figure 3c). For the roots, there was a significant treatment effect difference (*p* < 0.001; Figure 3d) with increased root partitioning in the JA-treated vines compared with the control. There was an increase in carbon partitioning to the roots immediately after JA treatment, with root partitioning resuming the initial rate within 24 h. No carbon tracer was detected in the apical shoot (not shown). That tissue was above the labelled leaf, where several more mature leaves would have been supplying the shoot with un-labelled carbon.

## 3. Discussion

The aim of this work was to investigate how the exogenous application of jasmonates on mature Chardonnay grape leaves affects the photoassimilation and export of carbon from the leaf and distribution to the developing grape berries. Understanding this response is important, because jasmonates play a critical role in defence activation, and exogenous chemical application of jasmonates is now being considered as a candidate to prime defence mechanisms for pest control in horticultural systems. Much of the work to date focuses on non-vine plants, with Populus, Arabidopsis, barley, conifers, and rice all represented in the literature, with very little on vines with growth and behavioural attributes specific to climbing plants.

Local exogenous application of MeJA to a mature leaf of Chardonnay induced resistance to botrytis in the treated leaf but not in untreated leaves on the same plant (Figure 2). Furthermore, the young leaves in both the control and MeJA-treated plants had much larger lesions than the mature leaves, suggesting that the ability of these young leaves to resist infection is much less than that of mature leaves, consistent with the idea of age-related resistance [26] and the accumulation of defence compounds as plant tissue ages [27]. As with the leaves, there was no evidence of a systemic response in the grape bunch. Berry total phenolic compounds and the key enzymes associated with a defence response (POX, CAD, and PAL) [23,24,28] all remained similar to the amounts in the control vines when the mature leaves were treated with JA (Table 1).

Any defence response is likely to use resources and affect partitioning. Four applications of MeJA applied weekly to the leaves and sampled just six weeks from the first MeJA application reduced the dry weight of both the shoots and roots, but the reduction was significant only for the roots (Figure 1). The reduction in root growth and root/shoot dry weight biomass may be a consequence of jasmonate-mediated regulation of growth through cell elongation and division. Experiments using Arabidopsis and rice demonstrate a reduction in cell number and cell elongation in the presence of JA [29]. Our grapevine shoots were pruned to one growing shoot (i.e., limited to one or two active vegetative growing points), whereas the roots were left unpruned, with numerous growing meristems and, consequently, a greater impact on growth. The reduction in total biomass accumulation may also be due to the effect of jasmonates on the photosynthetic system. In our work, JA applied to a mature leaf reduced its photosynthetic rate for at least 25 h. The effect of jasmonates on photosynthesis has been well documented and is due to a reduction in stomatal conductance [30,31,32,33,34] in addition to photo-inhibitory damage to the photosystem II [11], although the plant response has been shown to be dose dependent [35].

Carbon-11 radioisotope tracer experiments provided more detail of the carbon partitioning responses of the vine after jasmonate application. The treatments used JA rather than MeJA because of the risk that MeJA, being volatile, could contaminate the gas exchange equipment, compromise our control treatments from cross-contamination, and jeopardise future work in that laboratory. The function and behaviour of the jasmonates are closely linked within the plant. MeJA is the methyl ester of JA, and MeJA applied exogenously is de-esterified in the presence of the enzyme methyljasmonate esterase to JA within the plant. As a key step in the jasmonic acid pathway associated with plant defence, the endogenous JA has also been shown to be converted to MeJA in the presence of JA carboxyl methyltransferase [9,36,37].

The application of JA to mature source leaves reduced the export fraction of recently fixed photosynthate from the treated leaf (Figure 3). That reduction in carbon export is a likely consequence of an elevated disease resistance, as seen for example in the reduced *B. cinerea* infection after we applied MeJA (Figure 2). It seems that leaves had retained carbon to support the energy needed for elevated resistance. This strategy of reducing carbon export from treated source leaves is consistent with the idea of “high sugar resistance” where successful plant defence depends on available resources at the infection site [19].

Of the carbon exported following JA treatment, a greater portion was preferentially transported to the roots, showing that roots had a higher priority than other sinks. Such a change in carbon partitioning between sinks due to reduced leaf export can be explained by a mechanistic model of phloem transport, where sinks have different unloading kinetics [38,39]. Previous carbon tracer work found strong evidence that eliciting a plant defence response through exogenous jasmonate application can promote the flow of carbon to sink tissues: with barley, JA application to leaves increased carbon partitioned to the roots [13], and in Populus, there was an increase in both stems and roots [12]. JA applied to one-half of the root system of barley resulted in an increase in partitioning of available carbon to the untreated root half and no change for the treated portion [14]. This partitioning of resources away from the infection site to storage has been suggested as a strategy to shield carbon resources from predators [12]. Consistent with this hypothesis is the increase in berry sugar observed seven days after the second of two applications of JA (Table 1). At the time of JA application, root partitioning increased, while bunch partitioning did not change, despite the increase in berry sugar that we observed seven days after the treatments. In this dynamic system where sugar distribution can respond in many ways, the increased berry sugar may have used carbon remobilised from storage, such as in roots or stems, which become stronger sinks following leaf JA treatment.

Priming plant defence systems through stimulating defence-signalling pathways either directly through application of endogenous plant defence compounds or through environmental manipulations is likely to have a role in future integrated fruit production management systems. In terms of resource partitioning, the cost/benefit relationship is complex, requiring an understanding of the defence response and the effects on growth and productivity. In this work, we demonstrated that jasmonate application to mature source leaves of Chardonnay grapes reduces the export of recently fixed photoassimilate, allowing the leaf to maintain sufficient resources to support the defence response to *B. cinerea* infection. Our results highlight the importance of the balance of carbon partitioning for growth and resistance. These results are consistent with the theory that in some plant systems, carbon is preferably partitioned to the roots and other storage sites immediately following JA application, for later remobilisation to the fruit and other sinks [12,14].

## 4. Materials and Methods

Experiments were carried out on potted Chardonnay grapevines. An initial trial looked at the effect of foliar MeJA application on root and shoot biomass partitioning. A second trial examined the impact of foliar JA application on the plant defence response, photosynthetic rate, and carbon partitioning (using JA instead of MeJA to avoid contamination of the gas-exchange system). In the initial MeJA trials the vines held no fruit; in the JA experiments, the vines were pruned to one bunch subtending the oldest remaining leaf. Experiments were carried out at the Ruakura Research Station (Plant and Food Research, Hamilton, New Zealand) and, for carbon-11 radiotracer work, at IBG-2: Plant Sciences, Forschungszentrum Jülich, Germany.

### 4.1. Effect of Foliar MeJA Application on Biomass Partitioning

Mature leaves of the vines were treated with 4.5 mM MeJA (Sigma-Aldrich, an affiliate of Merck KGaA, Darmstadt, Germany) in 0.1% Tween^®^ 20 (polyoxyethylene sorbitan monolaurate; Sigma-Aldrich, an affiliate of Merck KGaA, Darmstadt, Germany) or a control solution of 0.1% Tween 20 in water. Treatments were applied using a small 1 L spray mister until runoff.

Sixteen treatment plants and sixteen control plants were grown outside with an automatic irrigation system. The vines were arranged in a block of 160 vines (eight rows of 20 plants each), well spaced with at least four buffer vines between each of the treated vines. Treatments were applied as foliar sprays four times at weekly intervals (4 December–15 January; southern hemisphere). Plants were then destructively harvested two weeks after the final treatment (29 January). These timings were selected to coincide with pre-bunch closure of adjacent vines that held a crop. Root, trunk, and shoot fresh weight were measured. The tissue was then dried at 80 °C for four days, and the dry weight was measured.

### 4.2. Effect of Foliar MeJA on Leaf Defence Response to Botrytis cinerea Inoculation

Vines were divided into two sets: one set of 15 vines had three mature leaves treated with 4.5 mM MeJA; the other 15 vines had three mature leaves treated with water + Tween as a control. The vines were then divided further into groups of five for inoculation with *B. cinerea*.

### 4.3. Botrytis cinerea Inoculum

*Botrytis cinerea* isolate BCK3 (originally isolated from kiwifruit) was maintained in cool storage at −70 °C in 15% glycerol (*v*/*v*) until required. Fresh cultures were grown on oatmeal agar (30 g ground oats, 20 g Bacto™ agar and 1 L of tap water) in the dark at 18 °C for 14 d. Conidia were harvested by flooding the cultures with sterile distilled water containing Tween 20 (0.01% *v*/*v*) and then filtering the subsequent suspensions through sterile cell strainers (Falcon, 100 µm mesh) and adjusting the concentration as required with the aid of a haemocytometer.

### 4.4. Leaf Inoculation

Three leaves per vine were inoculated: (1) the mature treated leaf; (2) a non-treated mature leaf, and (3) a non-treated young leaf. The leaves were lightly misted with sterile distilled water before placing four gamma-irradiated necrotic kiwifruit leaf disks (5 mm dia.) onto the adaxial surface of each grape leaf. The necrotic disks were positioned symmetrically approximately 2.5 cm apart with two on either side of the mid-rib vein and taking care to avoid placement over secondary veins. The necrotic disks were then inoculated with 7.5 µL of freshly prepared *B. cinerea* conidial suspension containing 1 × 10^5^ conidia/mL. Botrytis lesion diameters were measured after five days of incubation at 20 °C.

### 4.5. Effect of Foliar JA on Vine Metabolites and Defence Response

Seven vines were selected for each treatment (JA v control). Mature leaves of the vines were treated with 1 mM JA ( Sigma-Aldrich, an affiliate of Merck KGaA, Darmstadt, Germany) solution with 0.1% Tween 20 or a control solution of 0.1% Tween 20 in water two weeks and then again one week before sampling at pre-bunch closure (21 January 2021). Five berries were bulked together from a single bunch on each vine for analysis. Seven vines were used per treatment. Pre-bunch closure was selected, as it represents a time of significant import of carbon into the berries at a time of rapid fruit growth.

### 4.6. Sugar and Acid Analyses

Leaf and fruit tissues were frozen in liquid nitrogen then freeze dried and ground, and a subsample was extracted using 80% ethanol with the addition of adonitol and quinic acid as the internal standards for 1 h at 60 °C. Extracted samples were centrifuged and the supernatant decanted off. The residue was re-suspended in 80% ethanol re-spun and supernatants combined. The insoluble residue was put into Erlenmeyer flasks and analysed for starch, according to the method described by Smith et al. [40].

A subsample of the supernatant was taken, and the ethanol was evaporated using a steam of nitrogen gas. The sample was re-dissolved in ultra-pure water then passed through SP and QAE resin to remove amino and organic acids, respectively [41].

The eluent containing the sugars was collected, frozen, and freeze-dried. Samples were re-dissolved in ultra-pure water. Total sugars were analysed using a DIONEX ICS-3000 Reagent-Free™ IC (RFIC™) system with a CarboPac PA20 column (Thermo Fisher Scientific Inc., Waltham, MA, USA).

Organic acids were eluted from the QAE resin using 10% formic acid, frozen, then freeze-dried. Dried samples were re-dissolved in 10% iso-propanol, and a sub-sample was taken and placed in an auto sampler vial and dried, under vacuum over P_2_O_5_. Acids were derivatised with 1:1 (*v*/*v*) pyridine and *N*-methyl-*N*-(trimethylsilyl) trifluoroacetamide (MSTFA) at 60 °C for 15 min prior to analysis by gas chromatography using split injection and a DB 1701 30-m column with temperature programming from 130 °C to 270 °C over 35 min.

### 4.7. Total Phenolics and Defence Enzyme Assays

Grape berries were ground under liquid nitrogen in a mortar and pestle until they were the consistency of a fine powder.

For the extraction of total phenolics, 100 mg of ground tissue were placed in 1 mL 95% ethanol for two hours at room temperature in the dark. The samples were then spun down at 10,000 rpm for five minutes and the supernatant decanted into fresh Eppendorf tubes and stored at −80 °C until used. The pellet was retained and oven-dried at 45 °C for 48 h to obtain tissue dry weights.

Total phenolics were measured using a modification of the method described in Coseteng and Lee [42] based on increasing colour intensity of Folin–Ciocalteu reagent (Sigma-Aldrich, an affiliate of Merck KGaA, Darmstadt, Germany) with increasing phenolic concentrations. Stock samples of gallic acid standards were made up in 95% ethanol to concentrations of 0.2, 0.4, 0.6, 0.8, and 1 mg mL^−1^. Both the samples and standards were diluted 10-fold with water. Total phenolics were extracted in a solution containing 670 μL water, 67 μL sample/standard, and 133 μL Folin–Ciocalteu reagent, which was mixed thoroughly before adding 133 μL saturated sodium carbonate solution and mixing and was then left for 90 min in the dark. Then, 200 μL was transferred from each tube into a well on a 96-well multiwell plate, and the absorbance at 640 nm was read on a spectrophotometer plate reader (Biotek, Winooski, VT, USA). Each sample was read twice.

Enzyme assay extractions required 200 mg of tissue extracted in 1 mL of 0.5M K-PO_4_ extraction buffer containing 0.2 M ascorbic acid, 1.5% polyethylene glycol 4000, 50 mM cystine, 5 mM EDTA, and 0.4 M sucrose, adjusted to pH 8.8. Samples were extracted for 2 h on ice, spun down at 10000 rpm for 5 min, and the supernatant decanted into fresh Eppendorf tubes containing 20 mg insoluble polyvinylpyrrolidone. Samples were then vortexed and left a further 15 min before being spun down at 10,000 rpm for 5 min and the supernatant decanted into fresh Eppendorf tubes. A 120 μL aliquant of each sample was then put through a Pierce protein desalting spin column (product number 89849; Thermo Fisher Scientific Inc., Waltham, MA, USA). Both the crude and desalted samples were stored at −80 °C.

### 4.8. Phenylalanine Ammonia Lyase

Phenylalanine ammonia lyase (PAL) activity was measured using a modification of the method described in Bernards and Ellis [43] based on the ability of PAL to break down phenylanine to cinnamic acid and ammonia. A substrate was prepared containing 3.8 mL 0.1 M sodium tetraborate pH 8.8, 80 μL 1 mM unlabelled L-phenylalanine, and 80 μL L-[U-^14^C] phenylanine (Sigma-Aldrich, an affiliate of Merck KGaA, Darmstadt, Germany). Next, 50 μL of substrate was mixed with 50 μL of the desalted sample and incubated in a water bath at 40 °C for one hour before the reaction was stopped by adding 10 μL 4 N sulphuric acid. Then, 250 μL of 50mM cinnamic acid solution (in a 50/50 mix of toluene/ethyl acetate) was added to each sample and mixed thoroughly. The solution was centrifuged at 10,000 rpm for two minutes, 100 μL of the upper phase was removed to a scintillation vial, and the concentration of ^14^C labelled cinnamic acid was measured using a scintillation counter.

### 4.9. Peroxidase

Peroxidase activity was measured using a modification of the method described in Polle et al. [44]. A substrate mix containing 50 mM K-PO_4_ buffer pH 5.5, 40 mM guaiacol, and 10 mM H_2_O_2_ was prepared and equilibrated in a water bath at 40 °C for 30 min. An amount of 196 μL of substrate mix was added to 4 μL of sample, and the change in absorbance at 470 nm was measured over five minutes.

### 4.10. Cinnamyl Alcohol Dehydrogensase

An amount of 20 μL of sample was added to 180 μL of solution containing 100 mM Tris/HCl buffer pH 8.8, 100 μM conferyl alcohol, and 200 μM NADP. Cinnamyl alcohol dehydrogensase (CAD) activity was measured as the change in absorbance at 400 nm over 15 min.

### 4.11. Export and Partitioning of Recently Fixed Carbon and Photosynthetic Rate

We used two-year-old potted Chardonnay vines pruned to one shoot carrying a single bunch, with berries at pre-bunch closure. Vines were transported into the climate controlled room (22 °C, 16:8 light: dark period) and connected to the ^11^C-labelling system for 14–16 h prior to labelling, to ensure the plant had recovered from mechanical disturbance before starting the experiment. The mature leaf immediately apical to the grape bunches was selected as a major supply of carbon to the berries. An area of this source leaf of approximately 5 × 10^−3^ m^2^ was sealed into a Plexiglass™ chamber in a gas-exchange system that could be either a closed loop, with CO_2_ and humidity controlled at ambient, or open for standby overnight or to vent the radiotracer. Photosynthesis of the source leaf was monitored throughout the experiment. The gas loop was closed a few minutes before labelling the leaf with approximately 100 MBq ^11^CO_2_. The loop was opened 30 min later. Because carbon-11 has a short half-life of 20.4 min, radioactivity decays sufficiently by about 3 h after labelling to allow for repeated labelling and measurement. Here, a leaf was labelled five times at 5 h, 8 h, and 11 h into the light phase on day one, and at 5 h and 8 h into the light phase on day two, after which the next day’s plant was set up. JA or a wet control (0.1% Tween 20 and water) were applied one hour before the second labelling on day one. Counting equipment, with sensitivity-calibrated detectors arranged within radiation shielding, produced time-series of the tracer in the load leaf a(t) and sink shoot b(t), bunch c(t), lower stem d(t), and root e(t), after allowance for background and decay. Total mobilized tracer, mob, was defined as (mob = b + c + d + e). The various transport fractions were calculated from the 5 min means of the above, at *t* = 100 ± 2 min. The partitioning fraction for each sink was defined as its respective sink tracer as a fraction of the total exported tracer mob. Leaf export fraction was defined as total exported tracer, mob(t = 100), as a fraction of the tracer available for export from the load leaf. The latter, *lz0*, was estimated by linear extrapolation of the slowly reducing tracer a(t) back to the time of labelling. To show changes in a variate, its values were normalized to be relative to the initial pre-treatment value.

### 4.12. Statistical Analysis

Carbon partitioning: repeated measurements linear mixed models (ReML) were used to analyse the response variables export fraction, bunch partition, and root partition for the effects of treatment (control, 4 vines, and jasmonic acid, 6 vines) and time (3, 6, 24, and 27 h). A first-order antedependence correlation structure was used for the random effect of the plant. The data analysed were the ratios of the value for each time point divided through by the value at the initial time point prior to the treatment being applied. The data for bunch partition and root partition were log-transformed to meet the assumptions of normality and homogeneity. Repeated measurements linear mixed models (ReML) were used to analyse the response variable photosynthesis rate for the effects of treatment (control, 4 vines, and jasmonic acid, 4 vines) and time (1, 2, 3, 4, 5, 21, 23, 24, 25, and 26 h). A first-order antedependence correlation structure was used for the random effect of the vine. The standard errors from the mixed model analyses were used to construct 95% confidence intervals for each time and treatment combination for the four response variables of interest, and these confidence intervals were assessed as to whether the interval included the value 1 (the value of the ratio pre-treatment). If not, the treatment at that time point was deemed to be different to the pre-treatment value.

Defence compounds: One-way ANOVA was used to analyse the response variables phenolics, POX, CAD, and PAL for the effect of treatment (control vs. jasmonate, 7 vines each). The data for POX and CAD were log-transformed to meet the assumptions of normality and homogeneity.

Leaf botrytis: One-way ANOVA was used to analyse the response variable leaf botrytis for the effect of treatment (mature treated control, mature treated jasmonate, mature untreated control, mature untreated jasmonate, young treated control, and young treated Jasmonate, 5 leaves each).

Vine biomass: One-way ANOVA was used to analyse the response variable dry weight for the effect of treatment (shoot control, shoot jasmonate, root control, root jasmonate, stem control, and stem jasmonate, 16 observations each). One-way ANOVA was used to analyse the response variable root: shoot dry weight of treatment (control vs. jasmonate, 16 observations each).

Normality and homogeneity of the residuals were checked to justify the use of ANOVA. Fisher’s least significant differences was used post hoc to test for differences between the means at α = 0.05. The analyses were carried in Genstat 21st Edition (VSN International 2021).

## Figures and Tables

**Table 1:** Total soluble sugars, acid and phenolic concentrations (in µg of gallic acid equivalent per mg dry weight), and peroxidase (POX), cinnamyl alcohol dehydrogenase (CAD), and phenylalanine ammonia lyase (PAL) activity of Chardonnay grape berries sampled in January, one week after two foliar treatments of 1mM jasmonic acid (JA) applied one week apart. *n* = 7 vines, with 5 berries per vine combined.

Content	JA (Mean ± SE)	Control (Mean ± SE)
Total sugars mg g DW^−1^	771 ± 133	521 ± 66
Malic acid mg g DW^−1^	872 ± 162	1015 ± 132
Tartaric acid mg g DW^−1^	122 ± 6.3	126 ± 3.8
Citric acid mg g DW^−1^	12 ± 0.5	8.7 ± 3.4
Total phenolics µgGAE mg DW^−1^	511 ± 71	439 ± 45
POX ΔOD min^−1^ mg DW^−1^	204 ± 111	39 ± 23
CAD ΔOD min^−1^ mg DW^−1^	0.92 ± 0.63	4.2 ± 2.4
PAL CPM mg DW^−1^	190 ± 75	119 ± 61

## Data Availability

The data presented in this study are available on request from the corresponding author (N.G.).
